# Unenriched skin microbiota transplantation for cats: new road story for treating feline atopic skin syndrome

**DOI:** 10.29374/2527-2179.bjvm002823

**Published:** 2023-12-02

**Authors:** Kerem Ural, Hasan Erdoğan, Songül Erdoğan, Cansu Balıkçı

**Affiliations:** 1 Veterinarian, Aydın Adnan Menderes University, Faculty of Veterinary Medicine, Aydın, Turkey; 2 Veterinarian, Aydın Adnan Menderes University, Faculty of Veterinary Medicine, Aydın, Turkiye Aydın, Turkey

**Keywords:** dermatology, microbiome, cat, allergic diseases

## Abstract

Manipulation of skin biogeography has been the subject of study by the present authors for a very long while. Previous description and report identified the benefical application of skin microbiota transplantation (SMT) by the same researcher group, whom described unenriched skin microbiota transplantation at clinical veterinary practice for the first time among dogs. This study to our knowledge again for the first time reported herein aimed to investigate Un-smt application for treatment of feline atopic skin syndrome (FASS). This novel treatment intervention was performed similarly to previous description and methodology by use of Nivea Refining Clear-Up Strips (Ni-RcUs) either in autologue or heterologue route. Clinical biomarker for detecting the efficacy of Un-smt via Ni-RcUs evolved epidermal corneometric analytes (i.e. epidermal hydration and pH), relevant clinical scores The Feline Dermatitis Extent and Severity Index (FeDESI) and Visual Analogue Scale (VAS pruritus) and clinical observations performed weekly, at least. Both FeDESI and VAS pruritus scores were changed in relationship with smt. Pre-treament day 0 FeDESI scores (median ± SE) (72.5 ± 9.34), were significantly (p=0.001) higher than scores on day 10 (13.5 ± 2.55) switching the severity of the disease in all cases. Besides day 0 VAS pruritus scores were 6.0 ± 0.49 (median ± SE) (prior to treatment), whereas owner VAS pruritus score was decreased to 2.0 ± 0.34 (median ± SE) significantly (p=0.001). There were no side effects attributable to treatment applications. All cases were monitored for 6 months after completion of treatment in which no recurrence was observed. As a preliminary conclusion with selected number of cats with FASS, Un-smt with Ni-RcUs should be novel strategy for manuplating skin microbiome with treatment success.

## Introduction

The nomenclature “feline atopic syndrome” (FAS) refers to a broad spectrum of allergic disorders in cats, which includes allergic skin reactions, asthma/respiratory disorders, and gastrointestinal issues tied to hypersensitivities to allergens and foods (Favrot et al., 2014; Hobi et al., 2011; Santoro et al., 2021). Unlike in humans and dogs, the skin manifestations of FAS in cats are more varied, often presenting as miliary dermatitis, head and neck pruritus, self-induced alopecia, and eosinophilic changes (Favrot et al., 2014; Hobi et al., 2011; Santoro et al., 2021).

The cat microbiome (Ganz et al., 2022; Marsilio et al., 2019; Wernimont et al., 2020) has frequently been at the center of selected, but growing body of evidence, researches within the last few years, in particular as a candidate subject for novel clinical interpretation. The microbiota colonized at numerous anatomical locations are beneficial for multiple physiological/metabolic processes of both human and pet animal hosts. Besides growing body of literature denoted association between disrupted composition and functionality of the microbiota (Ganz et al., 2022; Marsilio et al., 2019; Wernimont et al., 2020) and multiple pathological issues. The latter supported a principle for advantageous stimulation of the microbiome. As a novel intervention being explored for influencing the microbiota among disease condition is microbiota (or its constituents) transferring from apparently healthy donors (Junca et al., 2022).

Until 2019, the cutaneous microbiota had not been determined using next-generation sequencing. Older et al. (2019) conducted an analysis on 69 cats, taking cutaneous samples from the axilla, dorsum, ear canal, nostril, and oral cavity, and subjected these samples to next-generation sequencing. This study revealed significant variations in alpha diversity of the skin microbiota across different cat breeds, with Sphynx and Bengal breeds exhibiting the most diverse communities. Several taxa were found to vary in abundance between cat breeds, notably *Veillonellaceae* and *Malassezia* spp. (Older et al., 2019). The cutaneous niche is inhabited by various bacterial communities specific to individual body locations (Older et al., 2017), while the fungal microbiome is more individual-specific to each cat (Meason‐Smith et al., 2017). Furthermore, the relative abundance of the feline cutaneous microbiota demonstrates greater diversity than previously identified through culture-based interpretation (Krogh & Kristensen, 1976).

Similar to canine (Cuscó et al., 2017; Pierezan et al., 2016; Rodrigues Hoffmann et al., 2014) skin, the vast majority bacterial phyla exhibited on cats are Proteobacteria, Firmicutes, Actinobacteria, and Bacteroidetes, through with differentiating proportions. Dissimilar to human skin, fundamentally inhabited by *Malassezia* spp. (Findley et al., 2013; Oh et al., 2014; Zhang et al., 2011), feline (Meason‐Smith et al., 2017) cutaneous niche are inhabited through a more diverse fungal mycobiota, with Dothideomycetes (the vast majority *Cladosporium* spp., *Alternaria* spp., *Epicoccum* spp.). On the other hand, given that the skin is colonized by several microorganisms, through the hypothesis that unbalanced microorganism inhabited could be related to disease condition, Older et al. (2017) investigated the cutaneous bacterial microbiota of both healthy and allergic cats. Genomic DNA extracted and the bacterial sequences among healthy cats exhibited alterations in species diversity and richness whereas bacterial phyla among allergic cat skin exhibited unique character belonging to the individual cat. The relative abundances of those bacterial species were altered among healthy and allergic skin, with *Staphylococcus* spp., in vast majority, was more abundant on allergic cat skin (Older et al., 2017). Given relative literature (Callewaert et al., 2021), the requirement for correction of cutaneous dysbiosis and the importance of natural treatment interventions, the aim of this study was to manipulate skin microbiome by use of Un-smt with Ni-RcUs against FASS.

## Material and methods

### Demographic data and laboratory evaluation

All 15 cats enrolled in the study were examined at the Department of Internal Medicine, Faculty of Veterinary Medicine, Aydin Adnan Menderes University, located in the Aegean Region of Turkey. The first author (K.U.) had long desire to establish a hypersensitivity laboratory, and all cases were evaluated at this facility. Written consent was obtained from all cat owners who participated in this study. The research was approved by the local ethics committee of Aydin Adnan Menderes University HADYEK, under approval number: 64583101/2017/053. The primary inclusion criteria for this study were cats presenting with FASS symptoms such as alopecia, head and neck pruritus with crusting, or miliary dermatitis. All cats underwent dermatological examinations and associated tests, including DermLite DL4 dermatoscopy, skin scraping, acetate tape impression smear, skin cytology, Polycheck IgE tests (with the Turkish distributor being the RDA Group, Istanbul), and epidermal corneometric analyses using the Callegari Soft Plus Device (Italy). Diagnoses were based on the aforementioned tests (Ural et al., 2018; Ural et al., 2019a, 2019b; Ural et al., 2021). Throughout the trial, all cats were maintained on a low carbohydrate diet (17% from Virbac Digestive commercial food). No medications were allowed, except for Triogermila I flk. (2x1 p.o.), as also described by the authors in another study (Ural et al., 2022a, 2022b).

Both tentative and presumptive diagnosis was coincided with diagnostic tree as described previously (Santoro et al., 2021). The latter algorithm involved novel terminology regarding FASS, establishing updated denotation of allergy, gastrointestinal and respiratory disorders among cats (Mueller et al., 2021). Under this classification FASS was dedicated to allergic dermatological issues in relationship with environmental allergy (Santoro et al., 2021; Ural et al., 2019a, 2019b; Ural et al., 2022a, 2022b; Ural et al., 2023). For detailed diagnosis of FASS, classification (Santoro et al., 2021) was performed in agreement with other data (Diesel & DeBoer, 2011; Favrot et al., 2014; Ravens et al., 2014). Given aforementioned data a total of 15 cats with FAS were presenting cutaneous manifestation were subclassified as flea allergic dermatitis (n=2), FASS (n=7) and feline cutaneous adverse food reaction (n=6), which were all enrolled at the present study.

**Figure 1:**
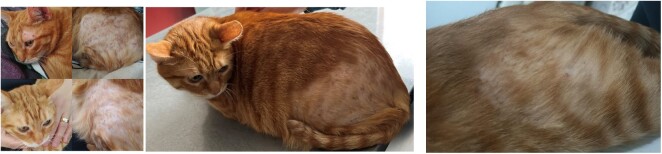
A cat with miliary dermatitis prior to and thereafter Un-smt on days 0., 7., 30 and 90. Soy bean was excluded from the diet as it was one of the culprit for hyperthyroidism related miliary dermatitis.

**Figure 2:**
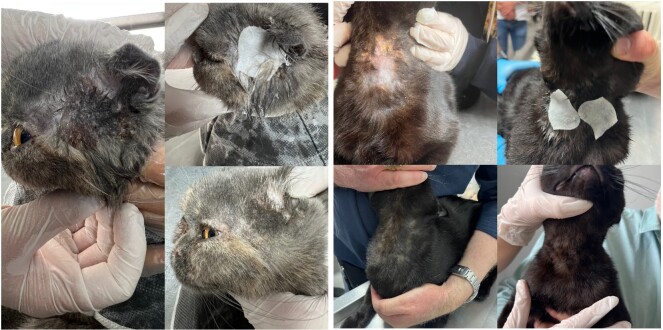
The first case on the left side was subjected to autologue Un-smt and the other relevant one on the right side with heterologue Un-smt. Both cases were recovered well and the before and after photos were recorded in 1-week apart period.

The vast majority of cats with FASS enrolled at this study presented alopecia, head and neck pruritus with crusting (Figures 1 and 2) or miliary dermatitis (Santoro et al., 2021).

### Unenriched skin microbiota transplantation procedure

Un-smt as a novel intervention involved 3 different sessions. I. session was composed of shaving of partial hair and exposing skin tissue. Preparation of donor in origin of 2 different methodology were available at the time of clinical practice. Depending on the availability of healthy donor, both methodology i) heterologous or ii) autologue origin of skin microbiota transplantation were applied. Decision was based on even if there was a healthy donor at the time of clinical application. Even if heterologous intervention was applied partial hair on skin with apparently healthy donor from a minimum of 4 different anatomical area with contrast composition (Ural et al., 2022a, 2022b; Ural et al., 2023) were shaved. If there was no donor at that time, diseased cat was transferred by its own skin microbiota as autologous intervention. Autologous methodology was preferred with 2 inclusion criteria [i.e. whether if there was no other healthy donor at the time of skin microbiota transplantation or partial lesion was evident onto the case with other relevant skin was deemed healthy as detected by cytology, tape stripping and dermatoscopical examination]. At least two healthy skin tissue were shaved with little hair removed. On II. occasion withdrawal of skin microbiome through Nivea Nivea Refining Clear-Up Strips (Ni-RcUs) was deemed available. Briefly copying skin microbiome from autologous or heterologous donor was performed as i) -unboxing Nivea Refining Clear-Up Strips (Ni-RcUs), ii) -moisturizing the skin with Lactated Ringers Solution and iii) placement of each Ni-RcUs on to the wet healthy skin with partial hair at least 4 different anatomical locations for a minimum of 10 minutes. On III. session (pasting) as a final step previously attached Ni-RcUs were peeled-off, then Ni-RcUs was suddenly transferred onto primary/secondary lesions periphery of diseased cutaneous involvement for 10 to 15 minutes [the clinician were allowed to moisten edges of the Ni-RcUs within Bepanthol Sensi Daily Plus Cream, if dried. Finally entire Ni-RcUs were removed out and smT procedure was finished.

### Scoring systems adopted

FeDESI was adopted herein, for scoring clinical signs (excoriations/erosions, erythema and self-induced alopecia) (Nuttall et al., 2004). On the other side the owners were instructed to assess pruritus through 10 cm visual analog scale (VAS) (Hill et al., 2007) which was described previously (Schmidt et al., 2012).

### Statistical analyses

The data of VAS pruritus and FEDESI scores of animals in pre- and post-treatment groups were tabulated as median and standard error. The comparison of scores between pre- and post-skin microbiota transplantation in animals was performed using the Mann-Whitney U test. Statistical analyses and graphs were conducted using GraphPad (9.5.1, Prism) software. Cases with a p-value less than 0.05 were considered statistically significant.

## Results

### Clinical scoring results

**Figure 3:**
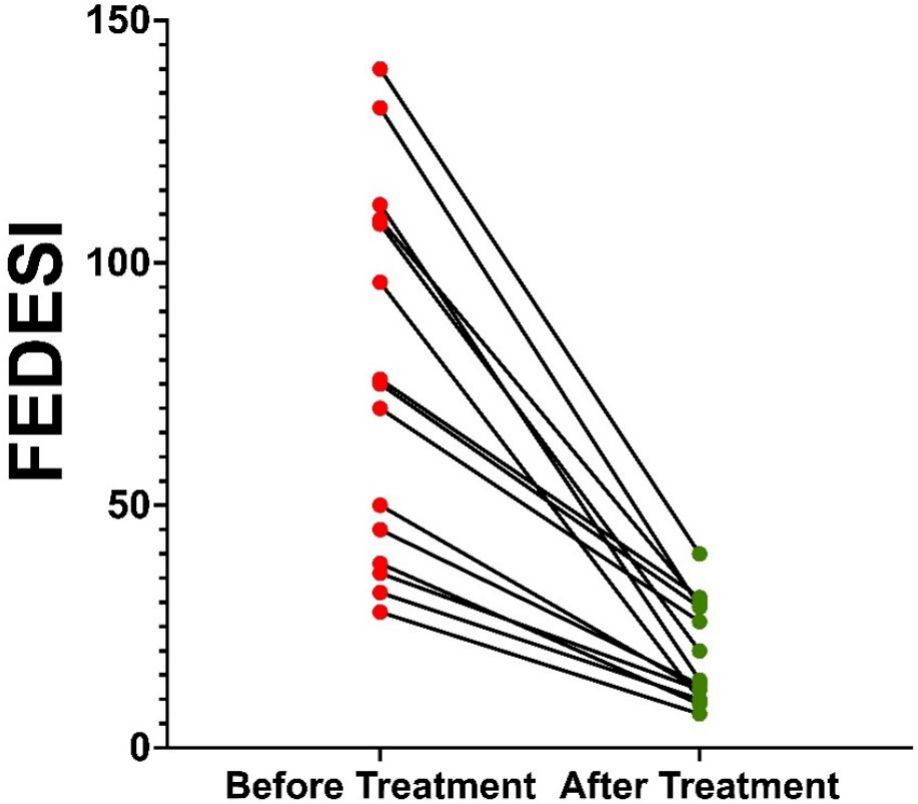
FEDESI scores, deemed most valuable scoring system used at this study with mean values prior to (in red color) and thereafter (in green color) smt application.

**Figure 4:**
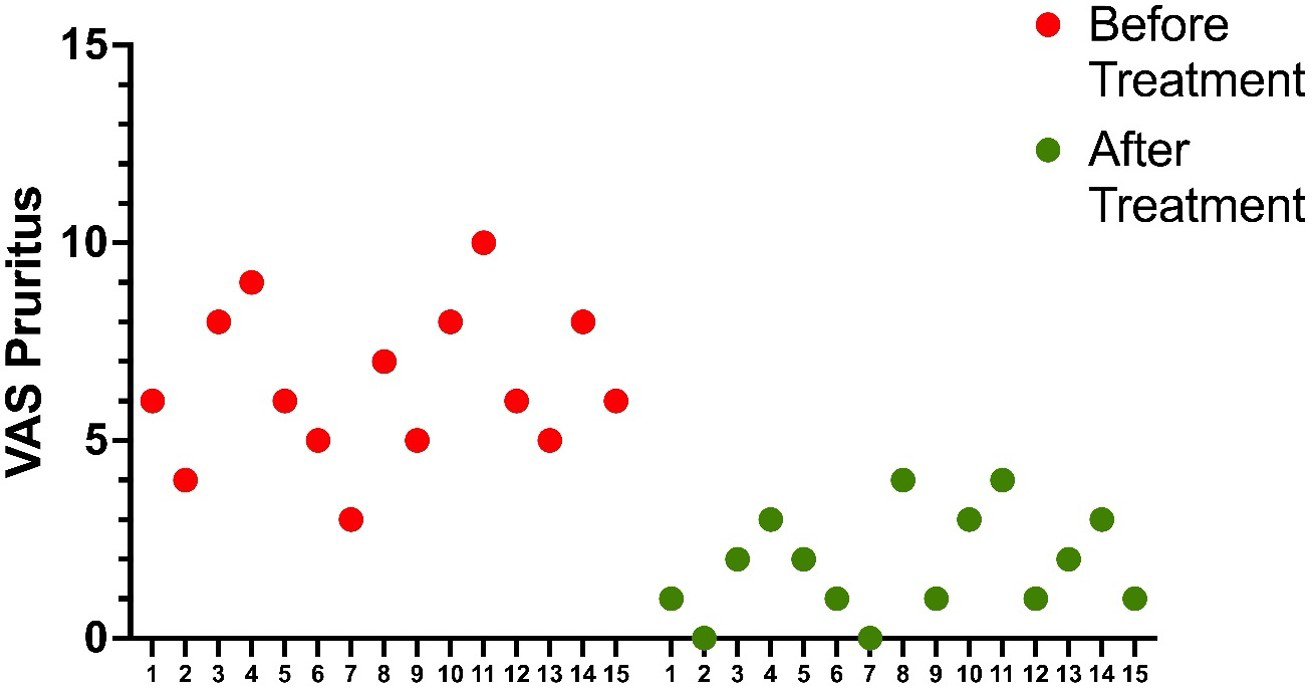
Individual distribution of VAS Pruritus Scores among cats enrolled prior to (in red color) and thereafter (in green color) smt application.

Both FeDESI (Figure 3) and VAS pruritus (Figure 4) scores were changed in relationship with smt. Pre-treatment day 0 FeDESI scores (median ± SE) (72.5 ± 9.34), were significantly (p=0.001) higher than scores on day 10 (13.5 ± 2.55) switching the severity of the disease in all cases. Besides day 0 VAS pruritus scores were 6.0 ± 0.49 (median ± SE) (prior to treatment), whereas owner VAS pruritus score was decreased to 2.0 ± 0.34 (median ± SE) significantly (p=0.001) (Table 1). Figures 3 and 4 showed FEDESI and VAS pruritus scores alterations before and after treatment. There were no side effects attributable to treatment applications. All cases were monitored for 6 months after completion of treatment in which no recurrence was observed.

**Table 1:** Statistical interpretation of VAs Pruritus and FEDESI Scores among cats enrolled.

	**Before Treatment** (*Median* ± *SE*)	**After Tretment** (*Median* ± *SE*)	***P value***
**VAS Pruritus**	6 ± 0.49	2 ± 0.34	0.001
**FEDESI**	72.5 ± 9.34	13.5 ± 2.55	0.001

SE: Standart Error

### Lesion distribution among cats

As was also aforementioned above at material and methods section regarding classification of disease condition, lesion distribution of cases prior to and thereafter smt was presented at Table 2.

**Table 2:** Cutaneous reaction patterns detected among cats with FASS prior to and thereafter Un-smt.

	**Prior to Un-smt -**	**After Un-smt**
alopecia	(n=14)	(n=4)
crusting	(n=8)	(n=2)
erythema	(n=5)	(n=0)
hyperpigmentation	(n=4)	(n=1)
Pruritus	(n=11)	(n=3)
Miliary dermatitis	(n=6)	(n=2)

Unenriched skin microbiota transplantation (Un-smt).

## Discussion

A pivotal challenge in microbiome transplantation research is identifying the specific components of the microbiome that confer therapeutic benefits. Recognizing these components could pave the way for developing treatments using these targeted combinations. Advances in omics technologies, next-generation sequencing, and bioinformatics have enriched our comprehension of microbiome composition and function (Costello et al., 2012; The Human Microbiome Project Consortium, 2012; Turnbaugh et al., 2007). Comparative analyses of microbiota between healthy and diseased individuals (Duvallet et al., 2017; Vital et al., 2017), including cats (Older et al., 2017), have facilitated the identification of certain microbiota components or functions that may be linked to disease states (Duvallet et al., 2017; Vital et al., 2017). In the present study to those of cats with FASS although we did not have the possibility for microbiome analysis (as because of financial deficiency), it should not be unwise to draw preliminary conclusion that cutaneous dysbiosis was evident, which prompted us to manipulate skin microbiome. In this study, regarding cats with FASS, even though we couldn't conduct a microbiome analysis due to financial limitations, it seems reasonable to infer a preliminary conclusion of evident cutaneous dysbiosis, leading us to consider interventions with the skin microbiome.

Given few data existing regarding canine skin microbiota, even much more fewer data is available about cats (Wernimont et al., 2020). In old fashioned but still needs to be taken into consideration study 10 healthy cats multiply sampled existed a predominance of *Micrococcus spp*., *Acinetobacter spp*. and streptococci, with fewer staphylococci (Krogh & Kristensen, 1976). On the other hand *Escherichia coli, Proteus mirabilis, Pseudomonas spp., Alcaligenes sp.* and *Bacillus sp.* were additionally detected rarely. In that study half of sampling sites did not exhibit any bacteria probably linked to the ‘cleanliness of the feline species’ (Krogh & Kristensen, 1976). However, but it is highly unlikely that those sites were indeed sterile, and the inability to isolate bacteria is probably from a combination of sampling methods, culture methods and lower overall bacterial abundance (Weese, 2013). As was also aforementioned above Older et al. (2017) reported altered relative abundances of relevant bacterial species among healthy and allergic skin, with *Staphylococcus* spp. abundancy on allergic cat skin (Older et al., 2017). At the present study we were unable to perform next generation sequencing (or other relevant diagnostic methodology) due to lack of financial support (as this was a self-funding project) for determining skin microbiome analysis. Moreover, we could not make speculation that skin dysbiosis were evident to those of cats enrolled, however previous findings could support that altered skin microbiota could participate (or as underlying etiology) to 15 cats with FAS at this study. Briefly this was an important notion that for why we performed Un-smt with Ni-RcUs. Moreover, if there was no evidence of cutaneous dysbiosis, we would be unable to reach those results we achieved at this study.

Even if disarranging microbiome is an activator or result of a disease, reconditioning or balancing the microbiome are strategies for therapeutical or preventive measurements (Weese, 2013). The customary intervention in an attempt to eliminate cutaneous infection in animals includes antimicrobials nearly everywhere. In materiality, abolishing cutaneous pathogen is as likely as not uncommon, since clinical and/or microbiological recovery are not the same. This may be briefly explained with major causes of bacterial invasion are the similar microorganisms frequently detected on healthy skin (Weese, 2013). Moreover, antibacterial treatment could thus be more suitably preferred by means of diminishing the choices of the offending agent for permitting the body’s own defenses and relevant components of the skin microbiota for suppression of the pathogen adequately. Other than widespread invasion of multidrug-resistant organisms (i.e., methicillin resistant staphylococci), where traditional antimicrobial usage could be of beneficial (Beck et al., 2012; Kania et al., 2004; Loeffler et al., 2007), the necessity for searching more knowledge about the commensal skin microbiota aroused questions whether such a nonspecific intervention is ideal. We herein at this study manipulated skin microbiota by Un-smt, thus could probably altered cutaneous niche and micro biogeography by this technique. Our subsequent study would thus be focused on microbiota analysis along with Un-smt technique.

Even if the core cutaneous microbiota of dogs is yet to be determined and its antimicrobial susceptibility exhibited, it may be claimed that frequently preferred antibacterials (i.e., for therapeutical armamentarium against pyoderma) might alter balance of the core microbiome and could eventually defeat both pathogenic and beneficial components (Weese, 2013). The probable participation of the skin microbiota in integumentary disorders (far off pyoderma) might prone thinking the idea of ‘optimization’ of the cutaneous niche as opposed to ‘battling’ pathogenic micro-organisms in a preferred fashion of nontargeted manner (Weese, 2013). By this context the present authors were unaware of finding documented reports on Un-smt among cats, which prompted us to perform this study.

At initial referral to our clinic all 15 cats were previously adminestered antibiotics without any treatment success, therefore skin echology manipulation was used. Moreover, by use of Ni-RcUs, we might probably have depleted pathogenic bacteria during transplantation to the donor, as because Ni-RcUs composed of citric acid. Same researcher group of this study, publishing another study (Ctrl X, Ctrl C and Ctrl V in Veterinary Dermatology: Splendiferous Era of Dermatology Art by Skin Microbiota Transplantation among Dogs with Cutaneous Adverse Food Reactions; under revision at the time of writing this manuscript) in which denoted that Ni-RcUs with its citric acid content could a) decorate superficial layers of old fashioned skin components (Ural et al., 2023), b) impede pseudomonas ceramidase (Inoue et al., 2010), c) diminish local pH in a similar manner to intestinal tract, as defined previously (Xue et al., 2023; Russel & Diez-Gonzalez, 1997) through declined colony of pathogens, via penetration to pathogenic cell wall consequently suppressed growth/reproduction (Xue et al., 2023; Russel & Diez-Gonzalez, 1997) and d) probably increase number of beneficial bacteria. The latter finding must be briefly discussed for better understanding of the readers. Beneficial (commensal or probiotic) bacteria could not be dominated by acidity, due to increased levels of potassium concentration (Gunal et al., 2006) through used citric acid, which able to influence bacterial cell cytoplasmic enzymes and transport systems arranging cells keep resistant to osmotic pressure (Cho & Finocchiaro, 2010). All aforementioned data should have been supported clinical recovery herein reported to those of cats with Fas.

As treatment biomarkers of origin, FEDESI and Vas Pruritus scores were deemed available at this study. Both FeDESI and VAS pruritus scores were altered in association with Un-smt. Prior to therapy with Un-smt on initial day 0 FeDESI scores (median ± SE) (72.5 ± 9.34), were significantly (p=0.001) higher than scores on day 10 (13.5 ± 2.55) switching the severity of the disease in all cats enrolled. Moreover day 0 VAS pruritus scores were 6.0 ± 0.49 (median ± SE) (prior to treatment), whereas owner VAS pruritus score was decreased to 2.0 ± 0.34 (median ± SE) significantly (p=0.001) (Table 1). As a well-known and validated scoring system composed of FEDESI, altered data supported clinical recovery due to local changes of cutaneous niche as was stimulated by Ni-RcUs. On the other side as was shown in Table 2 cutaneous reaction patterns were totally changed regarding alopecia, crusting, erythema, hyperpigmentation, pruritus and miliary dermatitis relevant to diagnosis. Strikingly number of cats with alopecia (14 vs. 4), erythema (5 vs. 0), crusting (8 vs. 2) and pruritus (11 vs. 3), prior to and thereafter Un-smt with Ni-RcUs, respectively, were cured. Changed cutaneous reaction patterns, as was shown on Table 2, also supported treatment success with Un-smt.

## Conclusion

It should not be unwise to draw preliminary conclusion that cat with FASS are capable of giving respond to Un-smt with Ni-RcUs in which manipulation of skin micro geography would be one of the future treatment choices in the therapeutic armamentarium.
